# Pricing Strategy of Construction and Demolition Waste Considering Retailer Fairness Concerns under a Governmental Regulation Environment

**DOI:** 10.3390/ijerph16203896

**Published:** 2019-10-14

**Authors:** Deng Li, Ying Peng, Chunxiang Guo, Ruwen Tan

**Affiliations:** 1 College of Architecture & Environment, Sichuan University, Chengdu 610065, China; 2018223055108@stu.scu.edu.cn (D.L.); tanruwen@scu.edu.cn (R.T.); 2 Business School, Sichuan University, Chengdu 610065, China; guochunxiang@scu.edu.cn

**Keywords:** C&D waste, closed-loop supply chain, game theory, fairness concern, governmental regulation

## Abstract

In order to investigate the issues of the recycling and remanufacturing of construction and demolition waste (C&D waste), this paper develops a closed-loop supply chain (CLSC) consisting of a manufacturer, a retailer, and a recycler, considering both the retailer’s fairness concern psychology and governmental regulations. Four mathematical models are developed for the calculations, and the models are solved through game theory. In both the decentralized and centralized scenarios, the members’ strategies are discussed and the optimal values of decision variables are determined. A numerical study is carried out for sensitivity analyses to verify the accuracy of the theoretical conclusions. The results reveal that retailer fairness concerns lead to a decrease in the wholesale price of building materials and negatively affect manufacturers’ profits. Additionally, governmental regulations can effectively increase the recycling amount and improve the utilization rate of C&D waste, and promote a virtuous cycle of the recycling and remanufacturing of C&D waste.

## 1. Introduction

With the rapid development of the world economy and the continuous improvement of people’s living standards, urbanization rates and industrialization processes are accelerating. More and more buildings and municipal infrastructure projects have to be built through the form of demolition, renovation, extension, and new constructions. Thus, the amount of construction and demolition waste (known as C&D waste) has been increasing year by year. For example, the annual C&D waste generated in Beijing has already reached 35 million tons [1]. If this waste cannot be properly disposed of, it will cause serious damage to the environment, adversely affect cultivated land, and threaten people’s physical and mental health. Therefore, the environmental and social problems caused by C&D waste are becoming more and more serious. The question of how to balance economic development and environmental protection is particularly important, and the effective implementation of C&D waste management is urgent.

In fact, many of the materials in C&D waste can be reused as renewable resources after being sorted, crushed, or eliminated. For example, abandoned masonry and waste concrete can be used as substitutes for sand to make building materials such as mortar, block, and brick after being crushed. Therefore, C&D waste has great potential for recycling and reuse. As the first country in the world to promote a circular economy, Germany was the first to propose the recycling and reuse of C&D waste [2]. Japan regards C&D waste as the “by-product” of construction, attaches great importance to its value as a renewable resource, and redevelops and utilizes it. In 1991, Japan enacted a law on resource reuse promotion [3]. In Europe, “C&D waste” constitutes a priority waste stream for waste management strategies due to the large volume and high recycling potential. Directive 2008/98/EC on waste stresses the need to improve the material recovery efficiency of C&D waste in the European Union [4].

As the largest developing country in the world, China is also suffering from problems of C&D waste. According to World Bank Data, China has become the world’s largest producer of solid waste, with about 2 billion tons of solid waste being C&D waste per year, i.e., about 40% of total urban waste. More than 400 cities are facing the phenomenon of a “garbage siege”. Finding a way to properly handle C&D waste has become a major problem facing China at present. Statistics show that the utilization rate of China’s C&D waste is extremely low, i.e., only 5%, compared with an average recycling rate of over 90% in developed countries and in regions such as Japan and the EU; there is a big gap. In the Regulations on the Management of Urban Construction Waste and Engineering Waste issued by the Ministry of Construction in 1996, the concept of C&D waste and its management was defined for the first time. Some Chinese cities, such as Chengdu and Yinchuan, have released legislations for punishing illegal waste dumping. Guangdong province has established a special management regulation for a C&D waste disposal management fee in 2013, while another Chinese city, Xi’an, has set up the baseline of a waste management fee for C&D waste in 2016. Meanwhile, there are also some supporting policies for the recycling of C&D waste. For example, some major Chinese cities, like Shenzhen and Kunming, have directly encouraged or invested social capital for the development of recycling facilities for C&D waste [5]. In 2015, China carried out the task of development and utilization of C&D waste in more than 20 cities, such as Beijing and Shanghai, and has already built about 20 production lines with an annual production capacity of over one million tons [6]. The government has also issued successive leading policies to provide support for C&D waste recyclers, such as the “Circular Economy Promotion Law of The People’s Republic of China and Regulations on The Management of Urban Construction Waste”. Some regions have even issued specific financial subsidy standards for C&D waste treatment enterprises or for remanufacturers of construction materials [7]. It can be seen that under the governmental regulations, China’s C&D waste management has taken a solid step forward, but there is still a long way to go.

Various problems concerning C&D waste management have also aroused widespread concern in academia recently. Many scholars have quantified and estimated the generation rate of C&D waste [8,9,10], and some have studied the applicability of emerging digital technologies such as big data and BIM (Building Information Modeling) in C&D waste management [11,12]. At the same time, focusing on the type of waste materials (such as concrete, asphalt, and brick) [13,14,15] and studying the waste material properties have also become mainstream directions of research. Although these scholars’ contributions to the management of C&D waste is obvious, there are still some questions which remain unsolved. In the construction field, the manufacturing of building materials and the recycling of C&D waste are two relatively independent processes. A lack of consideration of the entire life cycle leads to low recycling utilization rates of C&D waste. If we can’t deal with the problems from a systematic and broad perspective, recycling efficiency will be difficult to improve. Closed-loop supply chain management, however, is the design, operation, and control of a whole system to maximize value creation over the entire life cycle of a product, and can eventually motivate chain members to continue greening and improving sustainability. It’s a combination of forward and backward loop, and focuses on both the manufacturing and remanufacturing processes. Thus, we believe that the idea of closed-loop supply chain management is very suitable for C&D waste management. However, few scholars have optimized the C&D waste management mode from the perspective of closed-loop supply chain operation. Nowadays, the operational management of the manufacturing closed-loop supply chain is relatively mature. It can facilitate better information and material flows among chain members and improve the economic and environmental performance. If its advanced and excellent management experience and ideas are applied to the field of C&D waste management, it could be regarded as a new way to treat and deal with the aforementioned problems.

Throughout the literature on the behavior and decision-making of participants in closed-loop supply chains using game theory, scholars often regard participants as completely rational individuals aiming to pursue the maximization of their own interests. However, in real life, participants are not always completely rational, due to environmental, psychological, cognitive, and other reasons. For example, showing concern about fairness is one of the concrete embodiments of bounded rationality. Nobel Prize winners Samuelson and Sen have both pointed out that individuals are bound by self-interest, and often care about the behavior of other individuals [16]. Behavioral economics research finds that decision makers tend to have a strong sense of fairness concern; as the old Chinese saying goes, “inequality rather than want is the cause of trouble” [17]. For example, in 2013, the unit sales price of China’s wuchang rice was 199 yuan, while the grain farmers could only get 2 Yuan. This unfair phenomenon seriously affected the operation of the food supply chain in China [18]. A large number of behavioral experiments have shown that the fairness concern of decision makers is widespread and has a wide impact on social and economic activities [19]. Driven by the psychology of fairness concern, the individual may take some irrational action when he/she feels that he/she is being treated unfairly, which has a great impact on the decision-making processes of supply chain members [20]. Therefore, it is necessary to consider the participants’ fairness concerns when studying the decision making of closed-loop supply chain members.

In summary, in order to smoothly promote the process of C&D waste recycling management and continuously improve the utilization rate of C&D waste, it is of great research significance to study the closed-loop supply chain of C&D waste, considering both governmental regulations and retailers’ fairness concerns. Based on this, our goal is to gain managerial insights by exploring the following research questions:

- How do retailer fairness concern levels affect the closed-loop supply chain members’ behavioral decisions, profits, and the recycling amount of C&D waste?
- How does the governmental incentive mechanism affect the closed-loop supply chain members’ behavioral decisions, profits, and the recycling amount of C&D waste?
- What are the impacts of decentralized or centralized scenarios on decision variables?

In order to solve these problems, we have established decision models with retailer fairness concerns about governmental regulations. In decentralized and centralized scenarios, the optimal values of decision variables are gained. We have also analyzed the impact of the retailer’s fairness concern on supply chain members and the effectiveness of the governmental incentive mechanism. Our study aims to find the ideal management mode for the closed-loop supply chain of C&D waste and draw managerial insights.

## 2. Literature Review

This paper aims to address the issue of construction and demolition waste management from the perspective of a closed-loop supply chain operation, and perform a game-theoretical analysis for the pricing decisions of a closed-loop supply chain, considering both retailer fairness concerns and the role of the government. Thus, the related literature can be grouped into three streams: C&D waste management, the effect of fairness concern on supply chains, and governmental interventions on sustainable supply chains.

### 2.1. C&D Waste Management

“C&D waste” refers to a mixture of surplus materials generated from construction, renovation, and demolition activities [21]. It has many adverse effects on the environment, economy, and society, and has become a major challenge to sustainable development. Due to the limited landfill spaces, energy consumption, water pollution, and harmful gas emissions, C&D waste has already become a pressing issue in lots of countries [22]. Nowadays, the urgency of reducing, recycling, and reusing C&D waste has drawn great attention among both governments and the public.

Recently, the importance of C&D waste management has been increasingly adopted by academics, and many researchers have focused on it. Coronado, M. et al. (2011) [4] developed a two-step methodology for the quantification and management analysis of C&D waste and applied it to a case study in Cantabria, a northern Spanish region. Ajayi, S.O. et al. (2015) [23] explored factors impeding the effectiveness of existing C&D waste management strategies, as well as strategies for reducing the intensity of waste in the construction industry. Won, J. et al. (2016) [24] aimed to estimate the amount of construction waste prevented by a BIM-based design validation process based on the amount of C&D waste that might be generated due to design errors. Bilal, M. et al. (2015) [25] investigated the potential of BIM for C&D waste minimization and the applicability of big data technologies in the context of C&D waste minimization. Bilal, M. et al. (2016) [26] proposed the first Big Data-based architecture for C&D waste analytics which gives rise to a vibrant environment for design exploration and optimization to tackle C&D waste. Bian, B. et al. (2017) [27] proposed a method combining BIM technology with C&D waste management, and suggested that we can solve the problem of C&D waste utilization by using BIM technology to form an information attribute database. Nagapan, S. et al. (2013) [28] explored the impact of C&D waste on sustainable development, and formulated ways of avoiding or reducing it. They also highlighted a sustainable approach in managing the C&D waste.

The above studies are more related to the quantification and estimation of waste generation and the applicability of newly-emerging technologies in C&D waste management. However, none of them considered the entire life cycle of building materials, which is very important and cannot be ignored. In contrast to the aforementioned literature, we optimize the C&D waste management mode from the perspective of closed-loop supply chain operation; while considering the entire life cycle of building materials, we aim to eventually improve C&D waste recycling utilization rates.

### 2.2. The Effect of Fairness Concern on Supply Chains

Known as a manifestation of bounded rationality, fairness concern refers to a participant’s concern about inequalities among supply chain members. Many researches have indicated that a participant’s fairness concerns can significantly affect the supply chain members’ strategy, as well as the system’s profits.

Previous studies have provided valuable insights into how fairness concerns can potentially influence members’ strategies and profits, as well as channel cooperation and coordination. Qu, D.G. and Han, Y. (2013) [29] developed a two-echelon supply chain model considering manufacturers’ fairness concerns and analyzed their influence on the optimal channel product prices and profits. Chen, J.X. et al. (2017) [30] studied pricing and ordering issues in a dyadic supply chain and found that the members’ equilibrium strategies were significantly influenced by retailers’ fairness concern behavior. Wei, C.M. et al. (2017) [31] analyzed the influence of the channel members’ fairness concern preferences on the optimal decisions and the coordination status of supply chains. The theoretical results showed that members only benefit from fairness concerns when the level does not exceed a certain threshold value. Considering the fairness concerns of a service integrator, Liu, J. and Shu, S.L. (2015) [32] established a game model of a service provider and a service integrator. The results showed that with the increasing fairness concerns, the coordination flexibility will gradually weaken.

To our knowledge, studies on how to investigate the issue of fairness concerns in green or closed-loop supply chains via a game-theoretical framework are still limited. Han, X.H. et al. (2014) [33] studied pricing decisions in closed-loop supply chains. The results revealed that many participants exhibit bounded rationality and fairness concerns when they make decisions; furthermore, social relationships can also influence the degree of fairness concerns. Shi, P. et al. (2016) [34] investigated how fairness concerns and green efficiency may affect the degree product greenness and the profits of members. Motivated by a real business investigation, Du, B.S. et al. (2017) [35] analyzed a green supply chain model with sustainable green technology innovation development. The results indicated that fairness concerns can prompt supply chain members to invest more into sustainable green technology innovation development. Zhang, L.H. et al. (2018) [36] considered the impact of retailer fairness concerns on a green supply chain with one manufacturer and one retailer, and found that the retailer’s fairness concerns may cause a low green-degree of products and a profit loss for members.

Our work is different from theirs in two aspects. First, we consider the impact of fairness concerns in construction field and C&D waste management, which is absent from their paper. Second, we introduce governmental interventions and seek to discover the impact of fairness concerns on chain members’ strategies and profits, with and without governmental interventions.

### 2.3. Governmental Interventions on Sustainable Supply Chains

Growing awareness of environmental protection has led governments to put pressure on supply chains to produce and develop more eco-friendly and sustainable products. Nowadays, governmental decisions related to green supply chains have attracted considerable attention among researchers.

Qu, S.J. et al. (2019) [37] studied the optimal strategy for a green supply chain. Their results indicated that a higher governmental green subsidy provided to the manufacturer can lead to a higher level of social welfare, but interestingly, that a higher subsidy offered to the retailer may cause a lower level of social welfare. Ma, W.M. et al. (2013) [38] focused on how governmental consumption subsidies influence dual-channel closed-loop supply chains, and found that the subsidy is conducive to the expansion of closed-loop supply chains, and that both the manufacturer and the retailer are beneficiaries of the subsidy. Wang, K. et al. (2014) [39] found that governmental subsidies can incentivize remanufacturing activities, regardless of the remanufacturer’s channel choice, and that a suitable subsidy results in cooperation between the members of the supply chain.

Mohammadreza, S. and Morteza, R.B. (2018) [40] found that different governmental policies have significant impacts on the profit of the supply chain members, as well as on the environment. Madani, S.R. and Rasti-Barzoki, M. (2017) [41] discussed the determination of governmental tariffs in supply chain competition under government financial intervals. The results showed that the impact of raising subsidy rates is significantly greater than that of raising the tax rate, and additionally, that it leads to an increase in the sustainability of products and the profits of the government and of supply chains. Chen, W.T. and Hu, Z.H. (2018) [42] found that manufacturer behavioral strategies are influenced mainly by the governments’ strategy. The carbon taxes levied by governments have proved to be more effective than governmental subsidies to promote low-carbon manufacturing. Tan, Y. and Guo, C.X. (2019) [43] designed the “regulation menu” for governments. The results revealed that governmental policies can improve product recycling quality and remanufacturing technologies. The aforementioned studies have investigated and discussed governmental intervention, mainly in the manufacturing industry. Our paper differs from these studies because we consider governmental interventions in the construction field, and discuss the applicability of those regulations for C&D waste management.

To the best of the authors’ knowledge, none of the above studies has investigated C&D waste management from the perspective of closed-loop supply chain operation. Furthermore, few researchers have studied a closed-loop supply chain considering both fairness concerns and governmental interventions. Thus, the most important contribution of our research is to investigate C&D waste management from the perspective of closed-loop supply chain operation, and, at the same time to take both fairness concerns and governmental interventions into consideration. This paper also discusses supply chain member decisions and profits in centralized and decentralized models. Through our analyses, we aim to provide decision-making references for the government and enterprises in the construction industry.

## 3. Model Overview

### 3.1. Notation

*p*: Unit retail price of building materials, the decision variable of the retailer;

*ω*: Unit wholesale price of building materials, the decision variable of the manufacturer, *p* > *ω* > 0;

*b*: Unit buyback price of recycled materials, also the decision variable of the manufacturer;

*r*: Unit acquisition price for C&D waste from consumers, the decision variable of the recycler, *b* > *r* > 0;

*d*: Unit demand of building materials according to Ferrer, G. [44], d (p) = α − β × p, where α > 0 is the market capacity and β > 0 is the sensitivity of consumers to the selling price. To avoid negative demand, we assume that α − β × p > 0;

*η*: The difficulty coefficient of recycling C&D waste, indicating the comprehensive input cost of recycling;

*q*: The recycling amount of C&D waste. According to Yuan, X.G. and other scholars [45], *q* = γ + *r*, γ is the basic recycling amount of C&D waste;

C_T_: Fixed-asset investment cost of inspection, classification, and recycling of the recycler; refer to the similar method of Savaskan, R.C. et al. [46], C_T_ = η × q^2^;

C_t_: Unit cost of inspection, classification, and recycling of the recycler;

C_n_: Unit manufacturing cost of the manufacturer using new materials;

C_r_: Unit remanufacturing cost of the manufacturer using recycled materials, C_n_ > C_r_ + *b*;

Δ: The average saving cost from remanufacturing, Δ = C_n_ − C_r_ > 0;

*q*_0_: The standard recycling amount, *q*_0_ > 0;

ε: The reward and punishment degree for recycling, ε > 0;

ψ: The retailer fairness concern coefficient, indicating the level of fairness concern;

λ_1_, λ_2_: The retailer’s measure of fair profit of the manufacturer and the recycler, respectively;

π_R_, π_M_, π_T_: The profit of the retailer, the manufacturer, and the recycler, respectively;

U_R_, U_M_, U_T_: The utility of the retailer, the manufacturer, and the recycler, respectively;

π_g_^d^, π_g_^c^: The total profit of the supply chain system in decentralized or centralized scenarios with government intervention, respectively.

π_n_^d^, π_n_^c^: The total profit of the supply chain system in decentralized or centralized scenarios without government intervention, respectively.

### 3.2. Model Structure

In this study, both the economic and environmental aspects of sustainability are considered. We build a closed-loop supply chain containing a building materials manufacturer (manufacturer), a building materials retailer (retailer), and a third-party recycler (recycler). The recycler collects C&D waste from contractors or construction sites at an acquisition price, and recycles them professionally. After that, the recycled materials can be directly used for remanufacturing, and are sold to the manufacturer at a buyback price. The manufacturer first uses recycled materials for manufacturing, and when the recycled materials have been used up, he/she will choose to use new materials. The retailer is in charge of selling the building materials produced by the manufacturer. The retailer buys building materials from the manufacturer at a wholesale price and then sells them to consumers (construction contractors, etc.) at a retail price. Remanufactured building materials and new building materials are completely identical, and consumers have the same preference for the two kinds of building materials.

Research on behavioral economics shows that decision makers are not always rational-economic when making decisions, and that they often have a strong fairness concerns. Therefore, this paper considers that retailers have fairness concerns, that is, while focusing on his own profit maximization, he/she will also consider the profits of other participants and pay attention to the fairness of income distribution among the supply chain members. However, the retailer pays more attention to the unfair situation that is unfavorable to him/her, which is in line with the idea of “man struggles upwards” [47]. We assume that the retailer can accept his profit as λ_1_, λ_2_ times that of the manufacturer and the recycler, respectively [18], i.e., π_R_ = λ_1_ × π_M_, π_R_ = λ_2_ × π_T_. And if π_R_ < λ_1_ × π_M_ or π_R_ < λ_2_ × π_T_, the retailer will have feelings of jealousy, which will generate the negative effect of unfairness aversion, resulting in a utility loss of behavioral strategies. It is assumed that the rest of the participants are fairness neutral. A paper by Charness, G. [48] uses the following utility function to describe retailer fairness concerns, where ψ (ψ > 0) is a coefficient that indicates retailer fairness concerns, and the larger the value, the more the retailer values fairness in transactions:

(1) $$U_{R}=\pi_{R}-\frac{\psi }{2}\times (\lambda_{1}\times \pi_{M}-\pi_{R})-\frac{\psi }{2}\times (\lambda_{2}\times \pi_{T}-\pi_{R})$$

Given that the situation concerning C&D waste is not favorable, the Chinese government has adopted regulatory measures containing both the penalties and rewards, i.e., penalties for violations and supporting policies for the recycling of C&D waste [49,50]. Based on the original polices, we have made some expansions and improvement. It is assumed that the government implements a recovery reward–penalty mechanism for recyclers, with improving the amount of recycled C&D waste as a basic goal. The government determines the standard recycling amount, *q*_0_ (*q*_0_ > 0), and the reward and punishment degree for recycling, ε (ε > 0). Enterprises will be rewarded if they meet the standards set by the government, and will be punished if they fail to do so.

**Figure 1:**
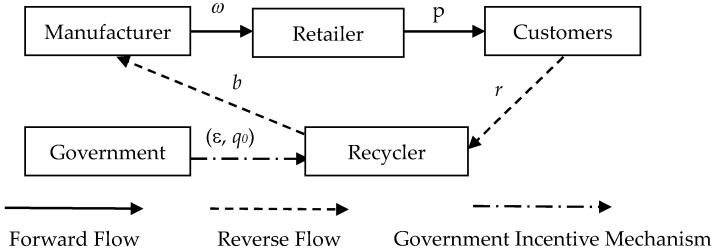
The graphical structure of a closed-loop supply chain under government.

The theoretical model structure of this paper is shown in Figure 1.

### 3.3. Key Assumptions

1. To simplify the model, we ignore the horizontal competition problem, and only consider the circumstances with one retailer, one manufacturer, and one recycler.

2. The manufacturer first uses recycled materials for manufacturing. When the recycled materials are used up, he/she will use new materials for manufacturing, and the average saving cost from remanufacturing will be Δ = C_n_ − C_r_ > 0, meaning that it’s profitable for the manufacturer to undertake remanufacturing activities;

3. Mehran Ullah et al. [51] proposed that the quality of remanufactured products is lower than that of new products. However, in the construction industry, there are many kinds of C&D waste, like waste concrete, scrap steel, and abandoned masonry, all of which can be remanufactured to the same quality as new materials. Referring to the research of Giutini, R. [52] and Biswajit Sarkar et al. [53], there is no difference in the function, quality, and performance of remanufactured building materials and new building materials, and consumers typically display no preference for either kind of building material.

4. Regardless of shortage and inventory issues, we only consider a single period setting in the closed-loop supply chain;

5. The basic purpose of the governmental incentive mechanism is to improve the amount of C&D waste recycling, regardless of the cost incurred by implementing the incentive mechanism.

## 4. Model Development

### 4.1. Closed-Loop Supply Chain Model without Governmental Intervention

No governmental intervention: In this case, recycling activity, production, and sales are not subject to governmental incentive mechanisms. We develop mathematical models considering the fairness concerns of retailers and obtain the optimal decisions of the members in decentralized and centralized scenarios. Their profit functions can be expressed as revenues − costs.

The expected profit of the retailer = sales revenue of building materials − the cost of purchasing of building materials.

The expected profit of the manufacturer = sales revenue of building materials − the cost of manufacturing − the cost of remanufacturing − the cost of purchasing recycled materials.

The expected profit of the recycler = sales revenue of recycled materials − the cost of recycling C&D waste − the cost of purchasing C&D waste.

Thus, the profit function of the retailer, the manufacturer, and the recycler will be as follows:

(2) $$\pi_{R}=(p-\omega )\times (\alpha -\beta \times p)$$

(3) $$\pi_{M}=(\omega -C_{n})\times (\alpha -\beta \times p)+(\Delta -b)\times (\gamma +r)$$

(4) $$\pi_{T}=(b-r-C_{t})\times (\gamma +r)-\eta \times {(\gamma +r)}^{2}$$

#### 4.1.1. Decentralized Scenario

In the decentralized scenario, a Stackelberg game framework is used, with the manufacturer as the leader, and both the retailer and the recycler as the followers. The manufacturer, the retailer, and the recycler make a sequential, non-cooperative, master-slave game with the goal of maximizing their own utility. According to Stackelberg game theory, this will be a two-stage game between the supply chain members. In the first stage, the manufacturer determines the wholesale price of building materials and the buyback price of recycled materials for the retailer and the recycler, separately. In the second stage, the retailer and the recycler make their best offers accordingly, that is, the retailer sets the retail price of building materials, and the recycler sets the acquisition price of C&D waste in response to a given wholesale price and a given buyback price. The manufacturer and the recycler don’t have fairness concerns, and they will only focus on their own profit maximization, so their utility functions are equal to their profit functions. The retailer has fairness concerns, which means that he/she will also consider other chain members’ profits and pay attention to the fairness of income distribution. The retailer’s utility function has already been discussed in Section 3. Thus, the game model in this case is as follows:

(5) $$\max U_{M}=\pi_{M}$$

(6) $$\max U_{T}=\pi_{T}$$

(7) $$\max U_{R}=\pi_{R}-\frac{\psi }{2}\times (\lambda_{1}\times \pi_{M}-\pi_{R})-\frac{\psi }{2}\times (\lambda_{2}\times \pi_{T}-\pi_{R})$$

According to the inverse induction method, the followers make their best response functions for their decision variables, which are expressed with the leader’s decision variables. Then, leader calculates the optimum values for his/her decision variables after replacing the followers’ decision variables by the response functions in his utility function. First, the concavity of the utility functions should be proven.

$$\frac{\partial^{2}U_{R}}{\partial p^{2}}=-2\times \beta \times (1+\psi )<0,\;\frac{\partial^{2}U_{T}}{\partial r^{2}}=-2-2\times \eta <0$$

Since the object functions are concave in nature, we can get the best response functions for unit prices by solving the first-order conditions simultaneously:

(8) $$p=\frac{2\times \alpha +2\times \alpha \times \psi +2\times \beta \times \omega +2\times \beta \times \psi \times \omega +\beta \times \psi \times \omega \times \lambda_{1}-\beta \times \psi \times C_{n}\times \lambda_{1}}{4\times \beta \times (1+\psi )}$$

(9) $$r=\frac{b-\gamma -2\times \gamma \times \eta -C_{t}}{2\times (1+\eta )}$$

Then, we replace net prices with these functions in the manufacturer’s utility function, and find that the object function is also concave in nature:

$$\frac{\partial^{2}U_{M}}{\partial b^{2}}=-\frac{1}{1+\eta }<0,\;\frac{\partial^{2}U_{M}}{\partial \omega^{2}}=-\frac{\beta \times (2+2\times \psi +\psi \times \lambda_{1})}{2\times (1+\psi )}<0$$

Then, the optimal values of the manufacturer’s decision variables will be obtained by solving the first-order conditions simultaneously:

(10) $$b_{n}{}^{d}=\frac{\left(\Delta +C_{t}-\gamma \right)}{2}$$

(11) $$\omega_{n}{}^{d}=\frac{\alpha \times (1+\psi )+\beta \times C_{n}\times (1+\psi +\psi \times \lambda_{1})}{\beta \times (2+2\times \psi +\psi \times \lambda_{1})}$$

Finally, the optimal values of the retailer and the recycler will be calculated by replacing these optimal values in the best response functions. After that, we can obtain the profits of the supply chain members and the total profit of the system:

(12) $$r_{n}{}^{d}=\frac{\Delta -3\times \gamma -4\times \gamma \times \eta -C_{t}}{4\times (1+\eta )}$$

(13) $$p_{n}{}^{d}=\frac{3\times \alpha +\beta \times C_{n}}{4\times \beta }$$

(14) $$\pi_{Rn}=\frac{{\left(\alpha -\beta \times C_{n}\right)}^{2}\times (2+2\times \psi +3\times \psi \times \lambda_{1})}{16\times \beta \times (2+2\times \psi +\psi \times \lambda_{1})}$$

(15) $$\pi_{Tn}=\frac{{\left(\gamma +\Delta -C_{t}\right)}^{2}}{16\times (1+\eta )}$$

(16) $$\pi_{Mn}=\frac{{\left(\gamma +\Delta -C_{t}\right)}^{2}}{8\times (1+\eta )}+\frac{(1+\psi )\times {\left(\alpha -\beta \times C_{n}\right)}^{2}}{4\times \beta \times (2+2\times \psi +\psi \times \lambda_{1})}$$

(17) $$\pi_{n}{}^{d}=\pi_{Rn}+\pi_{Tn}+\pi_{Mn}=\frac{3\times \left(1+\eta \right)\times {\left(\alpha -\beta \times C_{n}\right)}^{2}+3\times \beta \times {\left(\gamma +\Delta -C_{t}\right)}^{2}}{16\times \beta \times (1+\eta )}$$

(18) $$q_{n}{}^{d}=\gamma +r=\frac{\gamma +\Delta -C_{t}}{4\times (1+\eta )}$$

These are the optimal decision variables and profits when the game is balanced. Clearly, the wholesale price, the retailer’s profit, and the manufacturer’s profit are all influenced by retailer’s fairness concern coefficient. A more detailed discussion and an explanation of the optimal solutions may be found in Section 5.

#### 4.1.2. Centralized Scenario

In the centralized scenario, three decision-making individuals are vertically integrated. They act together as a single entity to make decisions cooperatively and simultaneously, in order to maximize the overall utility of the whole system. At this time, the decision variables of the system are the retail price of building materials and the acquisition price of the C&D waste. The wholesale price of building materials and the buyback price of recycled materials are the decision variables within the system, and they will not affect its overall utility. The utility function of the closed-loop supply chain will be as follows:

(19) $$\max U_{n}{}^{c}=\pi_{n}{}^{c}=\pi_{M}+\pi_{R}+\pi_{T}$$

Similarly, we can prove that the object functions are concave in nature. Then we can get the optimal values of those decision variables by solving the first-order conditions simultaneously:

(20) $$r_{n}{}^{c}=\frac{\Delta -\gamma -2\times \gamma \times \eta -C_{t}}{2\times (1+\eta )}$$

(21) $$p_{n}{}^{c}=\frac{\alpha +\beta \times C_{n}}{2\times \beta }$$

(22) $$\pi_{n}{}^{c}=\frac{\left(1+\eta \right)\times {\left(\alpha -\beta \times C_{n}\right)}^{2}+\beta \times {\left(\gamma +\Delta -C_{t}\right)}^{2}}{4\times \beta \times (1+\eta )}$$

(23) $$q_{n}{}^{c}=\gamma +r=\frac{\gamma +\Delta -C_{t}}{2\times (1+\eta )}$$

### 4.2. Closed-Loop Supply Chain Model with Governmental Intervention

In order to improve the recycling rate of C&D waste, and promote a green environment in the construction field, the government has implemented a recovery reward-penalty mechanism for recyclers. With improving the recycling amount of C&D waste as a basic goal, the government wants to encourage the recycler to learn and master advanced recycling technologies.

According to the recovery reward–penalty mechanism, we know that the total reward and punishment amount is:

(24) $$A(\varepsilon ,q_{0})=\varepsilon \times (q-q_{0})=\varepsilon \times (\gamma +r-q_{0})$$

Taking retailer fairness concerns into consideration, decentralized and centralized decision-making game models are established to obtain the optimal decisions of the members. The profit function of the retailer, the manufacturer, and the recycler will be as follows:

(25) $$\pi_{R}=(p-\omega )\times (\alpha -\beta \times p)$$

(26) $$\pi_{M}=(\omega -C_{n})\times (\alpha -\beta \times p)+(\Delta -b)\times (\gamma +r)$$

(27) $$\pi_{T}=(b-r-C_{t})\times (\gamma +r)-\eta \times {(\gamma +r)}^{2}+\varepsilon \times (\gamma +r-q_{0})$$

#### 4.2.1. Decentralized Scenario

In this case, the members make their own decisions independently to maximize their own utility. Similarly, a Stackelberg game framework is also used to solve the models. The game model used here is as follows:

(28) $$\max U_{M}=\pi_{M}$$

(29) $$\max U_{T}=\pi_{T}$$

(30) $$\max U_{R}=\pi_{R}-\frac{\varphi }{2}\times (\lambda_{1}\times \pi_{M}-\pi_{R})-\frac{\varphi }{2}\times (\lambda_{2}\times \pi_{T}-\pi_{R})$$

Using the aforementioned inverse induction method, we can prove the concavity of the utility functions and get the optimal values of the decision variables and the profits:

(31) $$b_{g}{}^{d}=\frac{\left(\Delta +C_{t}-\gamma -\varepsilon \right)}{2}$$

(32) $$\omega_{g}{}^{d}=\frac{\alpha \times (1+\psi )+\beta \times C_{n}\times (1+\psi +\psi \times \lambda_{1})}{\beta \times (2+2\times \psi +\psi \times \lambda_{1})}$$

(33) $$r_{g}{}^{d}=\frac{\varepsilon +\Delta -3\times \gamma -4\times \gamma \times \eta -C_{t}}{4\times (1+\eta )}$$

(34) $$p_{g}{}^{d}=\frac{3\times \alpha +\beta \times C_{n}}{4\times \beta }$$

(35) $$\pi_{Rg}=\frac{{\left(\alpha -\beta \times C_{n}\right)}^{2}\times (2+2\times \psi +3\times \psi \times \lambda_{1})}{16\times \beta \times (2+2\times \psi +\psi \times \lambda_{1})}$$

(36) $$\pi_{Tg}=\frac{{\left(\varepsilon +\gamma +\Delta -C_{t}\right)}^{2}-16\times \varepsilon \times (1+\eta )\times q_{0}}{16\times (1+\eta )}$$

(37) $$\pi_{Mg}=\frac{{\left(\varepsilon +\gamma +\Delta -C_{t}\right)}^{2}}{8\times (1+\eta )}+\frac{(1+\psi )\times {\left(\alpha -\beta \times C_{n}\right)}^{2}}{4\times \beta \times (2+2\times \psi +\psi \times \lambda_{1})}$$

(38) $$\pi_{g}{}^{d}=\pi_{Rg}+\pi_{Tg}+\pi_{Mg}=\frac{3\times \left(1+\eta \right)\times {\left(\alpha -\beta \times C_{n}\right)}^{2}+3\times \beta \times {\left(\varepsilon +\gamma +\Delta -C_{t}\right)}^{2}}{16\times \beta \times (1+\eta )}-\varepsilon \times q_{0}$$

(39) $$q_{g}{}^{d}=\gamma +r=\frac{\varepsilon +\gamma +\Delta -C_{t}}{4\times (1+\eta )}$$

#### 4.2.2. Centralized Scenario

In this case, the members make decisions together to maximize the overall utility of the whole system. The utility function of the whole closed-loop supply chain can be summarized as follows:

(40) $$\max U_{g}{}^{c}=\pi_{g}{}^{c}=\pi_{M}+\pi_{R}+\pi_{T}$$

Similarly, the concavity of the utility function can be proven. And we can get the optimal values of those decision variables by solving the first-order conditions simultaneously:

(41) $$r_{g}{}^{c}=\frac{\varepsilon +\Delta -\gamma -2\times \gamma \times \eta -C_{t}}{2\times (1+\eta )}$$

(42) $$p_{g}{}^{c}=\frac{\alpha +\beta \times C_{n}}{2\times \beta }$$

(43) $$\pi_{g}{}^{c}=\frac{\left(1+\eta \right)\times {\left(\alpha -\beta \times C_{n}\right)}^{2}+\beta \times {\left(\varepsilon +\gamma +\Delta -C_{t}\right)}^{2}}{4\times \beta \times (1+\eta )}-\varepsilon \times q_{0}$$

(44) $$q_{g}{}^{c}=\gamma +r=\frac{\varepsilon +\gamma +\Delta -C_{t}}{2\times (1+\eta )}$$

## 5. Model Analysis

In this section, the following conclusions and key findings are yielded by comparing the equilibrium solutions of decision-making models in four different scenarios.

**Proposition** **1.** The prices in each decision-making scenario have the following relationships:

(1) Neither the retail price nor wholesale price of building materials is affected by the government’s recovery reward–penalty mechanism: p_n_^d^ = p_g_^d^, ω_n_^d^ = ω_g_^d^, p_n_^c^ = p_g_^c^;

(2) The implementation of the government’s recovery reward–penalty mechanism will increase the acquisition price of C&D waste: r_g_^d^ > r_n_^d^, r_g_^c^ > r_n_^c^;

(3) The implementation of the government’s reward–penalty mechanism will reduce the buyback price of recycled materials: b_g_^d^ < b_n_^d^;

(4) The retail price of building materials in centralized scenarios is lower than in decentralized scenarios: p_n_^d^ > p_n_^c^, p_g_^d^ > p_g_^c^;

(5) The acquisition price of C&D waste in centralized scenarios is higher than in decentralized scenarios: r_n_^d^ < r_n_^c^, r_g_^d^ < r_g_^c^;

(6) A change in the retailer fairness concern coefficient doesn’t influence the acquisition price of C&D waste, the buyback price of recycled materials, or the retail price of building materials;

(7) The wholesale price of building materials is negatively correlated with the retailer fairness concerns level. When the retailer fairness concern coefficient goes up, the wholesale price of building materials goes down.

**Proof of Proposition** **1.**  

(1) By comparing the solutions of the two decentralized models, i.e., with and without government intervention, we obtain:

$$p_{n}{}^{d}=p_{g}{}^{d}=\frac{3\times \alpha +\beta \times C_{n}}{4\times \beta },\;\omega_{n}{}^{d}=\omega_{g}{}^{d}=\frac{\alpha \times (1+\psi )+\beta \times C_{n}\times (1+\psi +\psi \times \lambda_{1})}{\beta \times (2+2\times \psi +\psi \times \lambda_{1})}$$

And by comparing the solutions of the two centralized models, i.e., with and without government intervention, we obtain:

$$p_{n}{}^{c}=p_{g}{}^{c}=\frac{\alpha +\beta \times C_{n}}{2\times \beta }$$

(2) It can be proven by subtraction that:

$$r_{g}{}^{d}-r_{n}{}^{d}=\frac{\varepsilon +\Delta -3\times \gamma -4\times \gamma \times \eta -C_{t}}{4\times (1+\eta )}-\frac{\Delta -3\times \gamma -4\times \gamma \times \eta -C_{t}}{4\times (1+\eta )}=\frac{\varepsilon }{4\times (1+\eta )}>0,\;\mathrm{that}\;\mathrm{is}\;r_{g}{}^{d}>r_{n}{}^{d}$$

$$r_{g}{}^{c}-r_{n}{}^{c}=\frac{\varepsilon +\Delta -\gamma -2\times \gamma \times \eta -C_{t}}{2\times (1+\eta )}-\frac{\Delta -\gamma -2\times \gamma \times \eta -C_{t}}{2\times (1+\eta )}=\frac{\varepsilon }{2\times (1+\eta )}>0,\;\mathrm{that}\;\mathrm{is}\;r_{g}{}^{c}>r_{n}{}^{c}$$

(3) It can be proven by subtraction that:

$$b_{g}{}^{d}-b_{n}{}^{d}=\frac{\left(\Delta +C_{t}-\gamma -\varepsilon \right)}{2}-\frac{\left(\Delta +C_{t}-\gamma \right)}{2}=-\frac{\varepsilon }{2}<0,\;\mathrm{that}\;\mathrm{is}\;b_{g}{}^{d}<b_{n}{}^{d}$$

(4) It can be proven by subtraction that:

$$p_{n}{}^{d}-p_{n}{}^{c}=p_{g}{}^{d}-p_{g}{}^{c}=\frac{3\times \alpha +\beta \times C_{n}}{4\times \beta }-\frac{\alpha +\beta \times C_{n}}{2\times \beta }=\frac{\alpha -\beta \times C_{n}}{4\times \beta }>0,\;\mathrm{that}\;\mathrm{is}\;p_{n}{}^{d}>p_{n}{}^{c},\;p_{g}{}^{d}>p_{g}{}^{c}$$

(5) According to assumption γ > 0, Δ > C_t_, we obtain:

$$r_{n}{}^{d}-r_{n}{}^{c}=\frac{\Delta -3\times \gamma -4\times \gamma \times \eta -C_{t}}{4\times (1+\eta )}-\frac{\Delta -\gamma -2\times \gamma \times \eta -C_{t}}{2\times (1+\eta )}=-\frac{\gamma +\Delta -C_{t}}{4\times (1+\eta )}<0,\;\mathrm{that}\;\mathrm{is}\;r_{n}{}^{d}<r_{n}{}^{c},$$

$$r_{g}{}^{d}-r_{g}{}^{c}=\frac{\varepsilon +\Delta -3\times \gamma -4\times \gamma \times \eta -C_{t}}{4\times (1+\eta )}-\frac{\varepsilon +\Delta -\gamma -2\times \gamma \times \eta -C_{t}}{2\times (1+\eta )}=-\frac{\varepsilon +\Delta +\gamma -C_{t}}{4\times (1+\eta )}<0,\;\mathrm{that}\;\mathrm{is}\;r_{g}{}^{d}<r_{g}{}^{c}$$

(6) It can be proven by computing first-order derivatives that:

$$\frac{\partial r}{\partial \psi }=0,\;\frac{\partial b}{\partial \psi }=0,\;\frac{\partial p}{\partial \psi }=0$$

As shown, the acquisition price of C&D waste, the buyback price of recycled materials, and the retail price of building materials are not affected by the retailer fairness concern coefficient.

(7) Similarly, it can be proven by computing first-order derivative that:

$$\frac{\partial \omega }{\partial \psi }=\frac{-(\alpha -\beta \times C_{n})\times \lambda_{1}}{\beta \times {\left(2+2\times \psi +\psi \times \lambda_{1}\right)}^{2}}<0$$

As shown, the wholesale price is negative correlated with the retailer fairness concern coefficient. □

It may be seen from proposition 1 that the government’s reward–penalty mechanism only affects the values of optimal decisions in reverse flow, while the values of optimal decisions in forward flow are not affected. For the supply chain members, as a response to governmental intervention, the recycler will raise the acquisition price of C&D waste in order to increase the recycling amount. And due to the increase in the recycling amount, the manufacturer has to lower the buyback price of recycled materials to ensure his own profit. The implementation of the government’s reward-penalty mechanism will not only directly influence the recycler’s behavior, but also the manufacturer’s. In the centralized scenario, the retail price is lower and the buyback price is higher, that is, the sales volumes in forward flow and the recycling amounts in reverse flow both increase, which improves the efficiency of the closed-loop supply chain operation.

Retailer fairness concerns only affect the wholesale price of building materials. Because the retailer is only involved in the forward flow of the supply chain, his fairness concerns only affect the optimal decision prices in the forward flow. Furthermore, when the retailer has fairness concerns, he/she is more likely to exert pressure on stakeholders rather than change his own decision strategies. So, the wholesale price decreases, but the retail price remains unchanged.

**Proposition** **2.** The profits in each decision-making scenario have the following relationships:

(1) The overall profits of the supply chain in centralized scenarios are larger than the sums of the profit of each supply chain member in decentralized scenarios: π_n_^c^ > π_n_^d^, π_g_^c^ > π_g_^d^;

(2) The implementation of the government’s reward–penalty mechanism will not influence the retailer’s profit, but will lead to an increase in the manufacturer’s profit.

(3) The implementation of the government’s reward–penalty mechanism will increase the recycler’s profit and the sum of each supply chain member’s profit in the decentralized scenario, and the overall profit of the whole supply chain in the centralized scenario, when

$$q_{0}<\frac{2\times \gamma +2\times \Delta +\varepsilon -2\times C_{t}}{16\times (1+\eta )},\;q_{0}<\frac{6\times \gamma +6\times \Delta +3\times \varepsilon -6\times C_{t}}{16\times (1+\eta )},\;q_{0}<\frac{2\times \gamma +2\times \Delta +\varepsilon -2\times C_{t}}{4\times (1+\eta )}$$

is satisfied, respectively

(4) The retailer’s profit is positive correlated with his fairness concern coefficient, while the manufacturer’s profit is negative correlated with it. But the retailer fairness concern coefficient doesn’t affect the recycler’s profit, and interestingly, nor does it affect the sum of each member’s profit.

**Proof of Proposition** **2.**  

(1) It can be proven by subtraction that:

$$\pi_{n}{}^{c}-\pi_{n}{}^{d}=\frac{(1+\eta )\times {\left(\alpha -\beta \times C_{n}\right)}^{2}+\beta \times {\left(\gamma +\Delta -C_{t}\right)}^{2}}{16\times \beta \times (1+\eta )}>0,\;\mathrm{that}\;\mathrm{is}\;\pi_{n}{}^{c}>\pi_{n}{}^{d}$$

$$\pi_{g}{}^{c}-\pi_{g}{}^{d}=\frac{(1+\eta )\times {\left(\alpha -\beta \times C_{n}\right)}^{2}+\beta \times {\left(\varepsilon +\gamma +\Delta -C_{t}\right)}^{2}}{16\times \beta \times (1+\eta )}>0,\;\mathrm{that}\;\mathrm{is}\;\pi_{g}{}^{c}>\pi_{g}{}^{d}$$

(2) By comparing the retailer’s profit with and without government intervention, we obtain:

$$\pi_{Rg}=\pi_{Rn}=\frac{{\left(\alpha -\beta \times C_{n}\right)}^{2}\times (2+2\times \psi +3\times \psi \times \lambda_{1})}{16\times \beta \times (2+2\times \psi +\psi \times \lambda_{1})}$$

It is clear that the retailer’s profit is not changed. As for the manufacturer, we know from our assumption that Δ > C_t_, ε > 0, so γ + Δ − C_t_ > 0. Thus, we obtain:

$$\pi_{Mg}-\pi_{Mn}=\frac{{\left(\varepsilon +\gamma +\Delta -C_{t}\right)}^{2}-{\left(\gamma +\Delta -C_{t}\right)}^{2}}{8\times (1+\eta )}>0$$

That is to say, the manufacturer’s profit rises.

(3) By comparing the profits of the models with and without government intervention, we can determine that, when

$$\varepsilon +2\times \gamma +2\times \Delta -2\times C_{t}-16\times (1+\eta )\times q_{0}>0$$

is satisfied, that is, when:

$$q_{0}<\frac{2\times \gamma +2\times \Delta +\varepsilon -2\times C_{t}}{16\times (1+\eta )}$$

is satisfied, we always obtain:

$$\pi_{Tg}-\pi_{Tn}=\frac{{\left(\varepsilon +\gamma +\Delta -C_{t}\right)}^{2}-{\left(\gamma +\Delta -C_{t}\right)}^{2}}{16\times (1+\eta )}-\varepsilon \times q_{0}>0$$

when

$$3\times \varepsilon +6\times \gamma +6\times \Delta -6\times C_{t}-16\times (1+\eta )\times q_{0}>0$$

is satisfied, that is, when

$$q_{0}<\frac{6\times \gamma +6\times \Delta +3\times \varepsilon -6\times C_{t}}{16\times (1+\eta )}$$

is satisfied, we always obtain:

$$\pi_{g}{}^{d}-\pi_{n}{}^{d}=\frac{3\times {\left(\varepsilon +\gamma +\Delta -C_{t}\right)}^{2}-3\times {\left(\gamma +\Delta -C_{t}\right)}^{2}}{16\times (1+\eta )}-\varepsilon \times q_{0}>0$$

when

$$\varepsilon +2\times \gamma +2\times \Delta -2\times C_{t}-4\times (1+\eta )\times q_{0}>0$$

is satisfied, that is, when

$$q_{0}<\frac{2\times \gamma +2\times \Delta +\varepsilon -2\times C_{t}}{4\times (1+\eta )}$$

is satisfied, we always obtain:

$$\pi_{g}{}^{c}-\pi_{n}{}^{c}=\frac{{\left(\varepsilon +\gamma +\Delta -C_{t}\right)}^{2}-{\left(\gamma +\Delta -C_{t}\right)}^{2}}{4\times (1+\eta )}-\varepsilon \times q_{0}>0$$

(4) This can be proven by computing the first-order derivative. Whether governmental intervention exists or not, we always obtain:

$$\frac{\partial \pi_{T}}{\partial \psi }=0,\;\frac{\partial \pi_{M}}{\partial \psi }=-\frac{{\left(\alpha -\beta \times C_{n}\right)}^{2}\times \lambda_{1}}{4\times \beta \times {\left(2+2\times \psi +\psi \times \lambda_{1}\right)}^{2}}<0,\;\frac{\partial \pi_{R}}{\partial \psi }=\frac{{\left(\alpha -\beta \times C_{n}\right)}^{2}\times \lambda_{1}}{4\times \beta \times {\left(2+2\times \psi +\psi \times \lambda_{1}\right)}^{2}}>0,\;\frac{\partial \pi^{d}}{\partial \psi }=0$$

□

The threshold for the standard recycling amount, *q*_0_, that makes the total profit of the supply chain increase after the government’s reward-penalty mechanism is implemented, is 4/3 times larger in centralized scenarios than in decentralized ones. This indicates that even if the government raises the standard recycling amount to a certain extent, the members in centralized scenarios can still benefit from it, once again confirming the high efficiency of centralized scenarios. The government should pay more attention to the connection between the standard recycling amount, *q*_0_, and the reward and punishment degree for recycling, ε, when designing regulations. If the reward and punishment degree for recycling, ε, is small, but the standard recycling amount, *q*_0_, is large, the retailer’s enthusiasm cannot be fully mobilized by the incentive mechanism. It is difficult for the recycler to meet the recycling standards, and profit levels will diminish.

Since the recycler and the retailer share a common power status, there is no direct connection in their decision-making, so the recycler’s optimal decisions and profits are not affected by the retailer’s fairness concerns. And retailer fairness concerns force manufacturers to change their own decisions and transfer some of their profit to the retailer. As a result, the retailer’s profit increases and the manufacturer’s profit decreases, but the sum of their profits remains the same.

**Proposition** **3.** The implementation of the government’s recovery reward–penalty mechanism will increase the recycling amount of C&D waste. The recycling amounts of C&D waste in centralized scenarios are twice those in decentralized scenarios.

**Proof of Proposition** **3.** By comparing the recycling amount of C&D waste in the four models, we can show that:

$$q_{g}{}^{d}-q_{n}{}^{d}=\frac{\varepsilon }{4\times (1+\eta )}>0,\;q_{g}{}^{c}-q_{n}{}^{c}=\frac{\varepsilon }{2\times (1+\eta )}>0$$

(1) That is to say, the recycling amount of C&D waste in the models with governmental intervention is higher than that in the models without governmental intervention.

(2) When there is no governmental intervention, the recycling amount of C&D waste in the centralized and decentralized scenarios is as follows:

$$q_{n}{}^{c}=\frac{\gamma +\Delta -C_{t}}{2\times (1+\eta )},\;q_{n}{}^{d}=\frac{\gamma +\Delta -C_{t}}{4\times (1+\eta )},\;\mathrm{that}\;\mathrm{is}\;q_{n}{}^{c}=2\times q_{n}{}^{d}$$

(3) When governmental intervention exists, the recycling amount of C&D waste in the centralized and decentralized scenarios is as follows:

$$q_{g}{}^{c}=\frac{\varepsilon +\gamma +\Delta -C_{t}}{2\times (1+\eta )},\;q_{g}{}^{d}=\frac{\varepsilon +\gamma +\Delta -C_{t}}{4\times (1+\eta )},\;\mathrm{that}\;\mathrm{is}\;q_{g}{}^{c}=2\times q_{g}{}^{d}$$

We know that

$$q^{c}=2\times q^{d}$$

is always satisfied. □

**Proposition** **4.** As the remanufacturing average saving cost Δ rises, the acquisition price, *r*, the buyback price, *b*, the recycling amount, *q*, the manufacturer’s profit, π_M_, the recycler’s profit, π_T_, and the total profit of the supply chain, π, will increase. However, wholesale price, ω, retail price, *p*, and the retailer’s profit, π_R_, is fixed.

**Proof of Proposition** **4.** This can be proven by computing the first-order derivative. We only take the first scenario as an example, but other scenarios can be proven in the same way.

$$\begin{array}{c} \frac{\partial r}{\partial \Delta }=\frac{1}{4+4\times \eta }>0,\;\frac{\partial b}{\partial \Delta }=\frac{1}{2}>0,\;\frac{\partial q}{\partial \Delta }=\frac{1}{4+4\times \eta }>0,\;\frac{\partial \pi_{T}}{\partial \Delta }=\frac{\gamma +\Delta -C_{t}}{8+8\times \eta }>0,\;\\ \frac{\partial \pi_{M}}{\partial \Delta }=\frac{\gamma +\Delta -C_{t}}{4+4\times \eta }>0 \end{array}$$

$$\frac{\partial \pi }{\partial \Delta }=\frac{3\times (\gamma +\Delta -C_{t})}{8+8\times \eta }>0,\;\frac{\partial \omega }{\partial \Delta }=0,\;\frac{\partial p}{\partial \Delta }=0,\;\frac{\partial \pi_{R}}{\partial \Delta }=0$$

□

When the remanufacturing average saving cost rises, the manufacturer’s average manufacturing cost decreases and the marginal profit of the manufacturer increases. Since the saving cost comes from remanufacturing, the manufacturer is more willing to undertake remanufacturing activities to earn more. In order to have a better cooperation with the recycler, the manufacturer will raise the buyback price to transfer some of his/her profit to the recycler. And for the recycler, he/she will raise the acquisition price to increase the recycling amount of C&D waste, thereby increasing profits. As a result, the recycler’s profit and the manufacturer’s profit increase. However, the remanufacturing average saving cost is not directly related to the sales of building materials, so the wholesale price, the retail price, and the retailer’s profit stay the same. The total profit of the supply chain system increases.

**Proposition** **5.** The retailer’s profit is related to his/her measure of the fair profit of the manufacturer, λ_1_, but not to his/her measure of the fair profit of the recycler λ_2_. And when the retailer fairness concern coefficient, ψ, and other parameters remain unchanged, the wholesale price, ω, and the manufacturer’s profit, π_M,_ decrease as λ_1_ rises, while the retailer’s profit, π_R_, increases as λ_1_ rises.

**Proof of Proposition** **5.** This can be proven by computing first-order derivative. Since d = α − β × P > 0, and P > C_n_, we can know that:

$$\begin{array}{l} \frac{\partial \omega }{\partial \lambda_{1}}=-\frac{\psi \times (1+\psi )\times (\alpha -\beta \times C_{n})}{\beta \times (2+2\times \psi +\psi \times \lambda_{1}{)}^{2}}<0,\;\frac{\partial \pi_{M}}{\partial \lambda_{1}}=-\frac{\psi \times (1+\psi )\times {\left(\alpha -\beta \times C_{n}\right)}^{2}}{4\times \beta \times (2+2\times \psi +\psi \times \lambda_{1}{)}^{2}}<0,\;\\ \frac{\partial \pi_{R}}{\partial \lambda_{1}}=\frac{\psi \times (1+\psi )\times {\left(\alpha -\beta \times C_{n}\right)}^{2}}{4\times \beta \times (2+2\times \psi +\psi \times \lambda_{1}{)}^{2}}>0 \end{array}$$

□

The retailer believes that his/her profit is λ_1_ times that of the manufacturer, that is π_R_ = λ_1_ × π_M_. With the same level of fairness concern, when λ_1_ increases, the retailer believes that the ratio of the profit he/she deserves to the profit that the manufacturer deserves increases, resulting in a decrease in the wholesale price and the manufacturer’s profit, but an increase in the retailer’s profit when the game is balanced. The increase in λ_1_ severely hurts the interests of the manufacturer.

## 6. Sensitivity Analysis

The above section theoretically analyzes the impact of the retailer fairness concern level and governmental regulations on the decision-making of the closed-loop supply chain. In this section, a numerical study is carried out to examine the impact of various parameters on the model output. By using the Mathematica software for simulations, we could verify the accuracy of the aforementioned conclusions and explore the impact of the governmental regulations, the level of retailer fairness concern regarding members’ profits, and the recycling amount of C&D waste. The initial values of the various parameters are set as follows: α = 100, β = 10, η = 0.2, γ = 10, C_t_ = 5, C_n_ = 20, Δ = 12, ψ = 2, λ_1_ = 1.2, λ_2_ = 1.5, ε = 50, and *q*_0_ = 2.

### 6.1. Impact of the Retailer’s Fairness Concern on Profits

**Figure 2:**
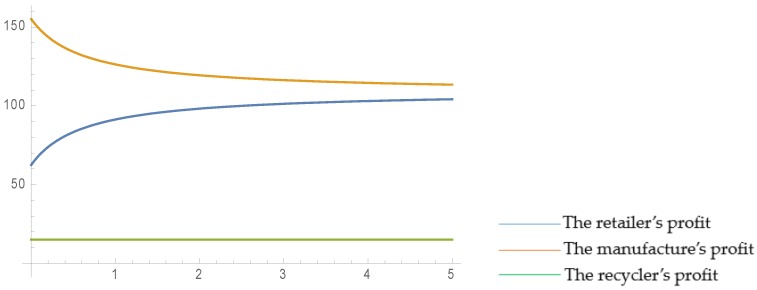
Effect of ψ on profits in closed-loop supply chain (CLSC) without government regulation.

**Figure 3:**
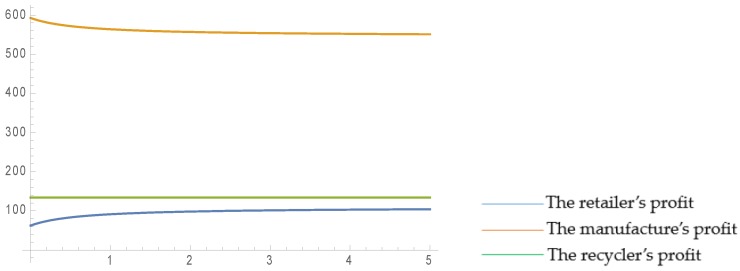
Effect of ψ on profits in CLSC with government regulation.

In order to vividly illustrate the relationship between the level of retailer fairness concern and the profit of each participant, we consider that the retailer’s fairness concern coefficient, ψ, varies within the range of 0–5, while the other parameters remain unchanged. The results are as follows:

As is shown in Figure 2 and Figure 3, when the retailer’s fairness concern coefficient increases, the retailer’s profit increases and the manufacturer’s profit decreases smoothly, but the recycler’s profit remains the same. This is because when the level of retailer fairness concern increases, the manufacturer has to transfer part of his/her profit to the retailer to achieve peaceful and harmonious coexistence with the retailer. Since there is no direct contact between the recycler and the retailer during the whole supply chain operation process, and the recycler only makes decisions based on the manufacturer’s behavior, the level of retailer fairness concern does not affect the recycler’s profit. In addition, the parameter settings satisfy:

$$q_{0}<\frac{2\times \gamma +2\times \Delta +\varepsilon -2\times C_{t}}{16\times (1+\eta )}$$

Comparing the two scenarios with and without governmental regulation, it can be found that when governmental regulations exist, although the retailer’s profit may be stable, the recycler’s profit obviously increases and the manufacturer’s profit significantly increases, which indicates that governmental regulation can benefit the members of the supply chain.

### 6.2. Impact of Governmental Regulation on Profits and Recycling Amount

In order to vividly illustrate the effect of the governmental regulations on the profit of each participant and the recycling amount of C&D waste, we keep other parameters unchanged and consider that the reward and punishment degree for recycling, ε, varies within the range of 0–350. (When ε = 0 is satisfied, it means no reward or punishment.) Then, the standard recycling amount, *q*_0_, varies within the range of 0–30. The results are as follows.

**Figure 4:**
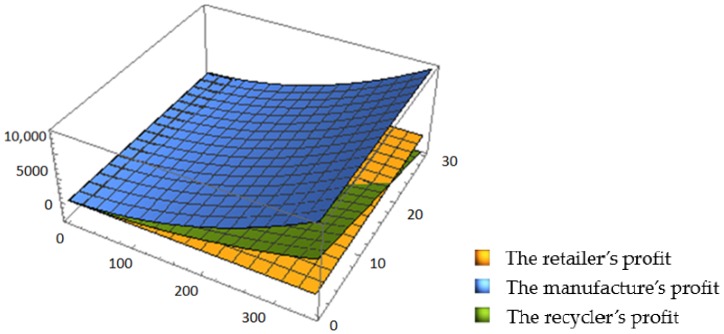
Effects of ε and *q*_0_ on each member’s profit in CLSC.

As displayed in Figure 4, the retailer’s profit is not affected by governmental regulations, and is fixed for all values of ε and *q*_0_. This is because the main target of the governmental reward–penalty mechanism is the recycler, and it therefore has no direct relationship with the retailer. Secondly, as the reward and punishment degree, ε, rises, the manufacturer’s profit rises rapidly, and a maximum is reached in the ε boundary. This is because the manufacturer produces both new and remanufactured building materials, and he/she can benefit more from the governmental incentive mechanism through coordinating the forward and reverse supply chain. It was also observed that the manufacturer achieves the highest profit, i.e., always higher than the retailer’s and the recycler’s, indicating that the manufacturer can transfer part of his/her profit to the recycler and the retailer by designing coordination contract mechanisms to mobilize the enthusiasm of the supply chain members and, thereby, to achieve a win-win situation. Furthermore, it can be concluded that the recycler’s profit increases with an increase of ε, but decreases with an increase of *q*_0_. Increasing ε increases what the government has to pay for tariffs when recycling amounts meet the standard, while increasing *q*_0_ means that the threshold with which that the recycler can be rewarded increases. As ε increases, the recycler’s profit increases smoothly, but as *q*_0_ increases, the recycler’s profit decreases at a steeper rate.

**Figure 5:**
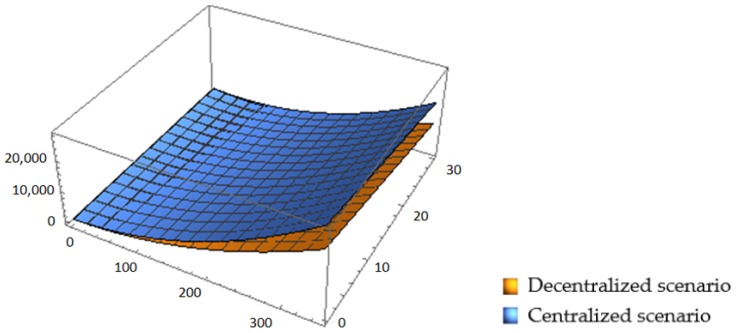
Effects of ε and *q*_0_ on the total profits in CLSC.

As illustrated in Figure 5, the total profit increases rapidly with an increase of ε, and decreases slowly with an increase of *q*_0_. In addition, for the same value of ε and *q*_0_, the total profit in the centralized scenario is always higher than that in the decentralized scenario, which means that choosing the centralized structure leads to more profit for the members. This is because the centralized structure both overcomes the double marginal effect and eliminates the negative effects of the retailer’s fairness concern.

**Figure 6:**
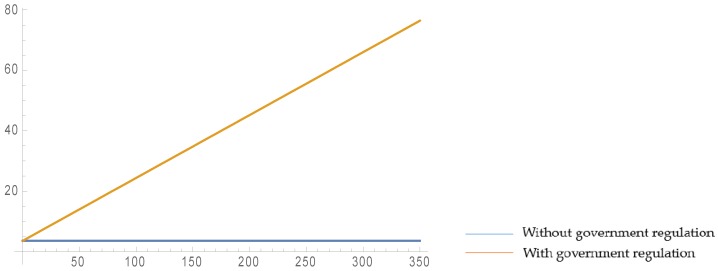
Effect of ε on the recycling amount in the decentralized scenario.

**Figure 7:**
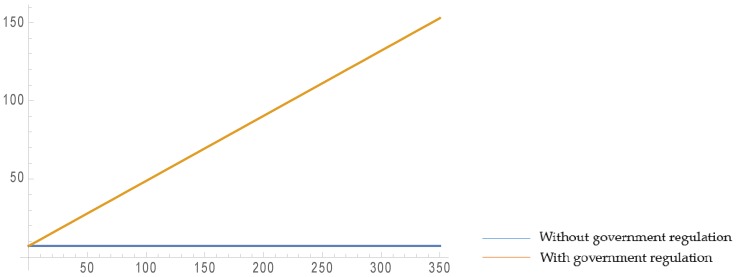
Effect of ε on the recycling amount in the centralized scenario.

Figure 6 and Figure 7 show that the recycling amount of C&D waste increases with an increase of ε. Compared with the decentralized scenario, the recycling amount in the centralized scenario is obviously higher, and also increases at a steeper rate. This shows that cooperation among the supply chain members leads to a growth in the recycling amount and to more sustainability, which once again proves the high efficiency of the centralized scenario.

## 7. Conclusions

Nowadays, the manufacturing of building materials and the recycling of C&D waste in the construction field are two relatively independent processes. The lack of consideration of the entire life cycle leads to the low recycling rates of C&D waste, which gives some insights that are useful in dealing with problems from a more systematic and global perspective. The closed-loop supply chain, however, is a combination of a forward loop and reverse loop, which means that chain members should coordinating both the forward and reverse material and information flows to maximize value creation. We believe that the idea of closed-loop supply chain management is very suitable for C&D waste management. Optimizing the C&D waste management mode, from the perspective of closed-loop supply chain operation, may become a research stream in the future.

In this paper, in order to improve the recycling efficiency of C&D waste and promote the process of C&D waste management, we study the behavior strategy problems of a closed-loop supply chain involving C&D waste. With the issues of the recycling, remanufacturing, and reuse of C&D waste as a basic goal, we build a closed-loop supply chain consisting of a retailer, a recycler, and a manufacturer, considering both the retailer’s fairness concern psychology and governmental regulations. The following competitive mathematical models were developed: a decentralized scenario without governmental intervention; a centralized scenario without governmental intervention; a decentralized scenario with governmental intervention; and a centralized scenario with governmental intervention. The members’ strategies in those four models are discussed and a numerical study is carried out for sensitivity analyses. Here, we may draw the following conclusions, which can provide managerial advice for governments and supply chain members.

The retailer’s fairness concern coefficient only affects the wholesale price of building materials. The wholesale price decreases continuously as the coefficient increases, resulting in a decrease in the manufacturer’s profit and an increase in the retailer’s profit. But the sum of their profits remains the same. Additionally, the recycler’s strategies are not affected by the retailer’s fairness concern coefficient, and the profit remains unchanged.

The government’s reward–penalty mechanism only affects the value of optimal decisions in the reverse flow, while the value of optimal decisions in forward flow is not affected. After the government implements the incentive mechanism for the recycler, the acquisition price of C&D waste increases, and the buyback price of recycled materials decreases. This means that more consumers are likely to collect C&D waste and sell them to the recycler. Thus, the recycling amount increases, and the manufacturer will prefer to use recycled materials instead of new materials for manufacturing to save money. We can conclude that the government’s recovery reward–penalty mechanism not only promotes the two processes of recycling and remanufacturing, but also that it increases the utilization rates of C&D waste. However, chain members do not always benefit from governmental regulation. They may face a profit loss when some thresholds are met. Thus, in order to encourage more enterprises to participate in C&D waste recycling management and maintain global sustainable development, the government should not only strengthen the recycling of C&D waste, but also pay attention to the chain members’ profits and make efforts to reduce the perceived losses.

Vertical cooperation among supply chain members overcomes the double marginal effect and eliminates the negative effects of the retailer’s fairness concerns. Therefore, in the centralized scenario, the retail price is lower and the buyback price is higher, which means an increase in both the sales volumes in the forward flow and the recycling amounts in the reverse flow. This improves the efficiency of the closed-loop supply chain operation and increases social welfare as well. Cooperation among members also leads to a higher recycling amount of C&D waste, which brings about substantial environmental improvements. Thus, supply chain members should take on social responsibility and choose to cooperate.

Although this paper has drawn some insights of managerial significance, and has contributed to the literature of C&D waste management, there are still some limitations. There is a certain deviation between reality and the assumptions made in this paper. First of all, this paper only considers one manufacturer, one retailer, and one recycler. Future research may consider multiple manufacturers, multiple retailers, and multiple recyclers, and establish a closed-loop supply chain network which is closer to reality. In this study, the governmental regulations are static. However, in reality, the nature of governmental regulations on C&D waste management is constantly changing. So, it is meaningful to establish a game model and analyze its operation mechanisms under a dynamic regulatory environment. Moreover, the question of how to design incentives for retailers, manufacturers, and recyclers to promote the process of the recycling of C&D waste is also a potential topic for future research. Studying the impact of incentive scheme types on system efficiency and member performance is also a good avenue of research. Furthermore, it is assumed that information is available to all members, which can be extended to asymmetric information settings. A final suggestion for further research is the consideration of a closed-loop supply chain led by the recycler or the retailer, rather than the manufacturer. It would be very interesting to include these factors into our model in the future.
